# Insulin-like growth factor-1 (IGF-1) levels in multiple sclerosis patients: A systematic review and meta-analysis

**DOI:** 10.1371/journal.pone.0297091

**Published:** 2024-04-17

**Authors:** Shirin Yaghoobpoor, Mobina Fathi, Kimia Vakili, Fatemeh Sayehmiri, Milad Alipour, Zahra sadat Miriran, Hani Ghayyem, Zohreh Tutunchian, Ramtin Hajibeygi, Zehra Batool, Moein Mirzadeh, Mohammad Hossein Aghazadeh, Mohammadreza Hajiesmaeili

**Affiliations:** 1 Student Research Committee, Faculty of Medicine, Shahid Beheshti University of Medical Sciences, Tehran, Iran; 2 Skull Base Research Center, Loghman Hakim Hospital, Shahid Beheshti University of Medical Sciences, Tehran, Iran; 3 Medical Student, Department of Medicine, Islamic Azad University Tehran Medical Sciences, Tehran, Iran; 4 Isfahan University of Medical Sciences, Isfahan, Iran; 5 School of Medicine, Shahid Beheshti University of Medical Sciences, Tehran, Iran; 6 Advanced Diagnostic and Interventional Radiology Research Center(ADIR), Tehran University of Medical Science, Tehran, Iran; 7 Department of Molecular Biology, Pasteur Institute of Iran, Tehran, Iran; 8 Dr. Panjwani Center for Molecular Medicine and Drug Research, International Center for Chemical and Biological Sciences, University of Karachi, Karachi, Pakistan; 9 Department of Surgery, School of Medicine, Shahid Beheshti University of Medical Sciences, Tehran, Iran; 10 Anesthesia and Critical Care Department, Critical Care Quality Improvement Research Center, Loghman Hakim Hospital, Shahid Beheshti University of Medical Sciences, Tehran, Iran; Hamad Medical Corporation, QATAR

## Abstract

**Background and objective:**

Multiple sclerosis (MS) is a chronic progressive autoimmune disorder of the central nervous system (CNS) that can cause inflammation, demyelination, and axon degeneration. Insulin-like growth factor-1 (IGF-1) is a single-chain polypeptide mainly synthesized in the liver and brain. IGF-1 causes neuronal and non-neuronal cell proliferation, survival, and differentiation. Therefore, it can be used in treating neuro-demyelinating diseases such as MS. The current systematic review and meta-analysis aims to compare the levels of IGF-1 in MS patients and healthy controls and also investigates IGF binding proteins (IGF-BP) and growth hormone (GH) levels between MS patients and healthy controls.

**Methods:**

In this study, we systematically searched electronic databases of PubMed, Scopus, Web of Science (WOS), and Google Scholar, up to December 2022. Studies that measured IGF-1, GH, IGFBP-1, IGFBP-2, or IGFBP-3 in MS patients and healthy controls in either blood or cerebral spinal fluid (CSF) were identified. We calculated Standardized mean differences (SMD) to compare levels of IGF-1, GH, IGFBP-1, IGFBP-2, or IGFBP-3 in MS patients and controls.

**Results:**

Finally, we included 11 eligible studies from 1998 to 2018. The sample size of included studies varied from 20 to 200 resulting in a total sample size of 1067 individuals, 531 MS patients, and 536 healthy controls. The mean age of the patient and control groups were 38.96 and 39.38, respectively. The average EDSS among patients was 4.56. We found that blood levels of IGF-1 (SMD = 0.20, 95% CI = -0.20 to 0.59, I2 = 82.4%, K = 8, n = 692), CSF level of IGF-1 (SMD = 0.25, 95% CI = -0.06 to 0.56, I2 = 0.0%, K = 3 n = 164) and blood levels of GH were not significantly higher in MS patients than controls (SMD = 0.08, 95% CI = -0.33 to 0.49, I2 = 77.0% K = 3, n = 421). Moreover, the blood levels of IGFBP-1 (SMD = 0.70, 95% CI = 0.01 to 1.40, I2 = 77%, K = 4, n = 255) were significantly higher in MS cases than in controls. However, the blood levels of IGFBP-2 (SMD = 0.43, 95% CI = -0.34 to 1.21, I2 = 64.2%, K = 3, n = 78) and blood levels of IGFBP-3 (SMD = 1.04, 95% CI = -0.09 to 2.17, I2 = 95.6%, K = 6, n = 443) were not significantly higher in patients than controls.

**Conclusion:**

Our meta-analysis revealed no significant difference in serum levels of IGF-1, GH, IGFBP-2, and IGFBP-3 between the MS group and healthy controls, except for IGFBP1. However, our systematic review showed that the studies were controversial for IGFBP-3 serum levels. Some studies found an increase in serum level of IGFBP-3 in MS patients compared to the healthy group, while others showed a decrease.

## Introduction

Multiple sclerosis (MS) is a chronic progressive autoimmune disorder of the central nervous system (CNS). Genetic and environmental factors have an etiological role in MS [1]. MS can cause inflammation, demyelination, and axonal degeneration. Also, it can lead to symptoms such as reduced mobility, pain, fatigue, and spasticity. It affects nearly one million people in the United States and results in an average lifetime caregiving cost of over four million dollars [2]. The incidence of MS is increasing due to earlier diagnosis, prolonged survival, and an increasing number of new cases [3].

Insulin-like growth factor-1 (IGF-1) is a single-chain polypeptide primarily synthesized in the liver. It is also produced in other tissues, including the brain, with its synthesis regulated by growth hormone (GH). Since IGF-1 cannot easily cross the blood-brain barrier, the brain is its primary source. IGF-1 production in the CNS, promotes the proliferation, survival, and differentiation of neuronal and non-neuronal cells [4]. It acts as a potent trophic factor for motor and sensory neurons and glial cells. IGF-1 has been shown to be a potent neuroprotective agent that supports the survival and production of myelin by oligodendrocytes. Therefore, it has potential in treating neuro-demyelinating diseases such as MS, however, the complex regulation of IGF-1 in the CNS is not yet fully defined [5].

Growth hormone (GH) is a nonglycosylated protein hormone produced in the anterior pituitary gland [6]. Its secretion is controlled by hypothalamic neurons that release stimulatory or inhibitory neuropeptides into the hypophyseal portal system, regulating GH synthesis and release by somatotropic cells. GH stimulates tissue and somatic growth by regulating cell division, regeneration, and proliferation. GH-responsive neurons are distributed throughout the entire CNS and have essential roles in the brain [7]. Evidence indicates that GH deficiency can result in a decrease in IGF-1 and insulin sensitivity, leading to memory deficits, fatigue, sleep problems, and attention deficit disorders [8]. Previous studies in mice showed that GH deficiency led to stunted neuronal growth, poor synaptogenesis, reduced locomotor activity, and a microcephalic cerebrum with hypomyelination due to glial cell proliferation cessation. These effects were reversed by GH replacement [9]. It has been demonstrated that GH treatment can impact neurogenesis, myelin synthesis, dendritic branching, and activate a subpopulation of neural stem cells that induce neurons in mice [10]. GH and IGF-1 are both growth factors with nutritional effects on neuronal regeneration in the CNS and the peripheral nervous system (PNS). They also participate in and stimulate protein synthesis in neurons, glia, oligodendrocytes, and Schwann cells aiding in neuron survival and inhibiting apoptosis. Thus, they may hold therapeutic potential for neuro-demyelinating diseases [11].

The current systematic review and meta-analysis mainly aimed at comparing the levels of IGF-1 between MS patients and healthy controls. We also compared the levels of IGF-binding proteins (IGF-BP) and GH between MS patients and healthy controls.

## Methods

This study was based on the PRISMA protocol for reporting systematic reviews and meta-analysis.

### Search strategy

We systematically searched online databases, including PubMed, Scopus, Web of Science, and Google Scholar up to December 2022. The search strategy incorporated the following keywords: "insulin-like growth factor", "insulin-like growth factor binding protein", "growth hormone", and "multiple sclerosis." There were no restrictions on the publication year. The reference lists of relevant papers were reviewed to ensure the inclusion of all relevant publications. The EndNote software was used for screening the searched studies. Furthermore, duplicate citations were subsequently removed, and unpublished studies were excluded.

### Inclusion criteria

Two authors (Z.T. and M.A.) independently conducted title-abstract and full-text screening. Controversies were resolved by the third author (Z.M.). The inclusion criteria consisted of the following: (1) observational studies with prospective, case-control, or cross-sectional designs. (2) studies that measured IGF-1, GH, IGFBP-1, IGFBP-2, and IGFBP-3. (3) studies that reported mean values and standard deviations (SDs) of the growth factor in both patient and healthy control groups or provided the required information for calculating these effect sizes.

### Exclusion criteria

We excluded letters, comments, short communications, reviews, meta-analysis, and ecological and animal studies. Additionally, studies without a control group and those that did not assess any of IGF-1, GH, IGFBP-2, or IGFBP-3 were excluded.

### Data extraction

Two investigators independently extracted data from each included study. The following information was extracted: name of the first author, publication year, individuals’ characteristics (mean age and sex), sample size (control and the patient group), mean levels and their SDs of IGF-1, IGFBP-3, IGFBP-2, and GH measured in blood and CSF in patient and control groups.

### Quality assessment

The quality of the studies included in this study was assessed using the Newcastle Ottawa Scale (NOS). This scale assigned a maximum of 9 points to each study based on the following parameters: 4 points for selecting participants, 2 points for comparability, and 3 points for assessing outcomes. A study with a score of 7–9 was considered as high quality, 4–6 had moderate quality, and 0–3 indicated a very high risk of bias. Two independent reviewers conducted the risk of bias assessment.

### Statistical analysis

This meta-analysis compared the levels of IGF-1, IGFBP-1, IGFBP-2, and IGFBP-3 in the blood and CSF of MS patients with a control group using Stata version 15 (Stata Corp, College Station, TX, USA). After data extraction, we conducted a meta-analysis to determine if there was sufficient data for each factor. Mean values and SDs of GH and IGF-1 in patient and the healthy control groups were utilized to calculate the overall effect sizes. The standardized mean difference (SMD) between patients and the control group was calculated as a unit of analysis for the assessed factors. To determine the overall effect size, we applied a random effects model that accounted for between-study variations. Heterogeneity was assessed using I2 statistics and Cochrane’s Q test. I2 value greater than 50% or a P-value less than 0.05 for the Q test indicated significant between-study heterogeneity. Sensitivity analysis was used to detect the impact of a specific study on the overall effect size. A formal test of Begg examined the possibility of publication bias, with a significance level set at a P-value less than 0.05.

## Results

### Study selection and characteristics

**Fig 1:**
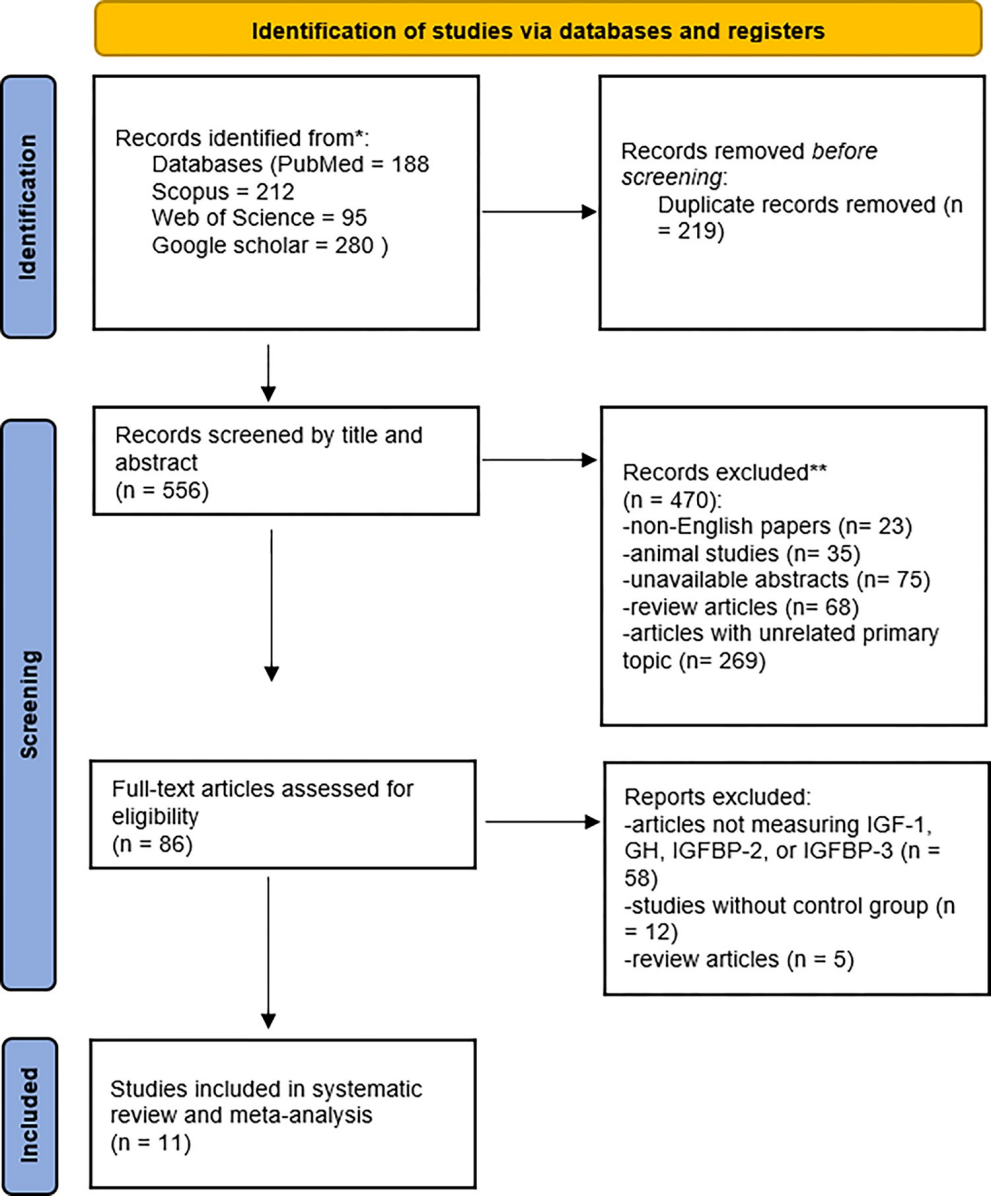
PRISMA flow diagram.

The current systematic review and meta-analysis study followed the PRISMA guidelines. From the initial 556 unduplicated studies resulting from the systematic search, 86 were excluded after title-abstract screening. The full text of the remaining articles was screened, and finally, 11 eligible studies were included in our systematic review and meta-analysis. For the screening process and reasons for exclusion, refer to Fig 1.

The included studies were conducted between 1998 and 2018 in various countries, including the Netherlands, Croatia, Finland, Egypt, Italy, Ireland, Italy, Kuwait, and Spain. The sample sizes of included studies ranged from 20 to 200, resulting in a total sample size of 1067 individuals, 531 MS patients, and 536 healthy controls. The mean age of patients and controls was 38.96 and 39.38, respectively. The average EDSS among patients was 4.56. Detailed characteristics of the included studies are presented in Table 1.

**Table 1:** Characteristics of the included studies. Abbreviations: HC, healthy control; MSD, Multiple Sclerosis; SD, standard deviation; CSF, Cerebrospinal fluid; N, numbers; EDSS, Expanded Disability Status Scale; IGF, insulin-like growth factor; IGFBP, Insulin-like growth factor binding protein; GH, growth hormone.

Author, Year	place	N (HC/MSD)	Age (mean ±SD) (HC/MSD)	EDSS (mean ±SD	Materials	Metabolites	Levels of measured metabolites in the patient (Mean ± sd)	Levels of measured metabolites in the control Mean ± sd	Key findings
**Nageeb,** **2018 [27]**	Egypt	46/46	33.19 (5.94)/35.11 (4.73)	4.57 ± 2.58	serum	IGF-1	IGF-1 = 138/37± 53/65 (ng/ml)	IGF-1 = 151/77±42/92 (ng/ml)	IGF-1 levels lower in the MSD group
**Akcali,** **2017 [12]**	Turkey	100/100	34.7 (9.5)/34.58 (10.5)		serum	GH IGF-1 IGFBP-3	GH = 1/91±0/34 (nmol/l) IGF-1 = 191±8/52(mg/ml) IGFBP-3 = 3/92±0/8(μg/mL)	GH = 1/79±0/36 (nmol/l) IGF-1 = 192±9/93(mg/ml) IGFBP-3 = 4/21±0/81(μg/mL)	GH levels higher in the MSD group IGF-1 levels lower in the MSD group IGFBP-3 levels are significantly lower in the MSD group
**Al-Temaimi,** **2017 [17]**	Kuwait	77/100	28 (8.14)/32 (8.88)	2 ± 1.75	plasma	IGFBP-1	IGFBP-1 = 11/09±13/61(ng/ml)	IGFBP-1 = 5/55±10/26(ng/ml)	IGFBP-1 levels were higher in the MSD group
**Gironi,** **2013 [13]**	Italy	62/64	48.87 (7.14)/50.21 (8.08)	4.57 ± 2.17	serum	GH IGF-1	GH = 0/87±1/32(ng/ml) IGF-1 = 152/8±55/4(ng/ml)	GH = 1/69±3/35(ng/ml) IGF-1 = 136/4±47/4(ng/ml)	GH levels lower in the MSD group IGF-1 levels were higher in the MSD group
**Lanzillo, 2011 [19]**	Italy	60/41	Range: 30–50		serum	IGF-1 IGFBP-3	IGF-1 = 237/47±103/53 (ng/ml) IGFBP-3 = 6769/66± 2027/80(ng/ml)	IGF-1 = 253/85±43/77 (ng/ml) IGFBP-3 = 4064/67± 882/65(ng/ml)	IGF-1 levels lower in the MSD group IGFBP-3 levels were higher in the MSD group
**Hosback,** **2007 [16]**	Ireland	10/10	Range: 28.6–73.1		serum	IGF-1 IGFBP-3 IGF-2 IGFBP-2 IGFBP-1	IGF-1 = 166/9±116/19 (ng/ml) IGFBP-3 = 4058±593 (ng/ml) IGF-2 = 878±91 (ng/ml) IGFBP-2 = 438±275 (ng/ml) IGFBP-1 = 40/6±22/9(ng/ml)	IGF-1 = 145/69±41/5 (ng/ml) IGFBP-3 = 4677±589 (ng/ml) IGF-2 = 989±158 (ng/ml) IGFBP-2 = 439±144 (ng/ml) IGFBP-1 = 46/1±21/6(ng/ml)	IGF-1 levels were higher in the MSD group IGFBP-3 levels were lower in the MSD group IGF-2 levels lower in the MSD group IGFBP-2 levels were lower in the MSD group IGFBP-1 levels lower in the MSD group
**Poljakovic,** **2006 [14]**	Croatia	49/46			blood	GH IGF-1	GH = 0/8±1/22 (mic.mol) IGF-1 = 31/62±9/22(mic.mol)	GH = 0/63±0/28(mic.mol) IGF-1 = 35/01±11/01(mic.mol)	GH levels higher in the MSD group IGF-1 levels lower in the MSD group
**Poljakovic,** **2006 [14]**	Croatia	49/46			CSF	GH IGF-1	GH = 0/43±0/33(mic.mol) IGF-1 = 3/4±0/87(mic.mol)	GH = 0/82±0/28(mic.mol) IGF-1 = 3/17±0/71 (mic.mol)	GH levels lower in the MSD group IGF-1 levels were higher in the MSD group
**Wilczak,** **2005 [24]**	Netherland	30/34	44 (9.7)/49.5 (8.7)	7 ± 0.96	serum	IGFBP-3	IGFBP-3 = 3/83±0/86 (pg/ml)	IGFBP-3 = 3/74±0/78 (pg/ml)	IGFBP-3 levels were higher in the MSD group
**Pirttila,** **2004 [32]**	Finland	25/14	47.5 (12.9)/32.4 (8.5)		CSF	IGF-1 IGFBP-2	IGF-1 = 0/44±0/18 (micg/l) IGFBP-2 = 142±39(micg/l)	IGF-1 = 0/4±0/11 (micg/l) IGFBP-2 = 149±37(micg/l)	IGF-1 levels were higher in the MSD group IGFBP-2 levels were lower in the MSD group
**Wilczak, 1998 [15]**	Netherland	15/15			serum	IGF-1 IGFBP-3 IGF-2 IGFBP-2 IGFBP-1	IGF-1 = 264±69/71(ng/ml) IGFBP-3 = 4460±1820/3(ng/ml) IGF-2 = 875±232/37 (ng/ml) IGFBP-2 = 463±240/12 (ng/ml) IGFBP-1 = 1/81±1/54 (ng/ml)	IGF-1 = 227±116/18 (ng/ml) IGFBP-3 = 4000±929/51(ng/ml) IGF-2 = 808±170/41 (ng/ml) IGFBP-2 = 444±220/76 (ng/ml) IGFBP-1 = 0/83±0/81 (ng/ml)	IGF-1 levels were higher in the MSD group IGFBP-3 levels were higher in the MSD group IGF-2 levels were higher in the MSD group IGFBP-2 levels were higher in the MSD group IGFBP-1 levels were higher in the MSD group
**Wilczak, 1998 [15]**	Netherland	15/15			CSF	IGF-1 IGFBP-3 IGF-2 IGFBP-2	IGF-1 = 0/95±0/81 (ng/ml) IGFBP-3 = 10/6±12/39 (ng/ml) IGF-2 = 19±7/74(ng/ml) IGFBP-2 = 241±69/71 (ng/ml)	IGF-1 = 0/9±0/5 (ng/ml) IGFBP-3 = 11/3±12/78 (ng/ml) IGF-2 = 20±7/74(ng/ml) IGFBP-2 = 201±54/22(ng/ml)	IGF-1 levels were higher in the MSD group IGFBP-3 levels were lower in the MSD group IGF-2 levels lower in the MSD group IGFBP-2 levels were higher in the MSD group
**Torres-Aleman, 1998 [18]**	Spain	13/15			serum	IGF-1 IGFBP-3 IGFBP-2 IGFBP-1	IGF-1 = 161/88±5/38 ng/ml IGFBP-3 = 3/35±0/04 mg/l IGFBP-2 = 696/62±16/86 ng/ml IGFBP-1 = 2/57±0/11 mic.g/l	IGF-1 = 147/08 ±4/04 ng/ml IGFBP-3 = 3±0/04 mg/l IGFBP2 = 674/15±19/67 ng/ml IGFBP-1 = 2/38±0/08 mic.g/l	IGF-1 levels were significantly higher in the MSD group IGFBP-3 levels were higher in the MSD group IGFBP-2 levels were higher in the MSD group IGFBP-1 levels were higher in the MSD group

#### IGF-1

**Fig 2:**
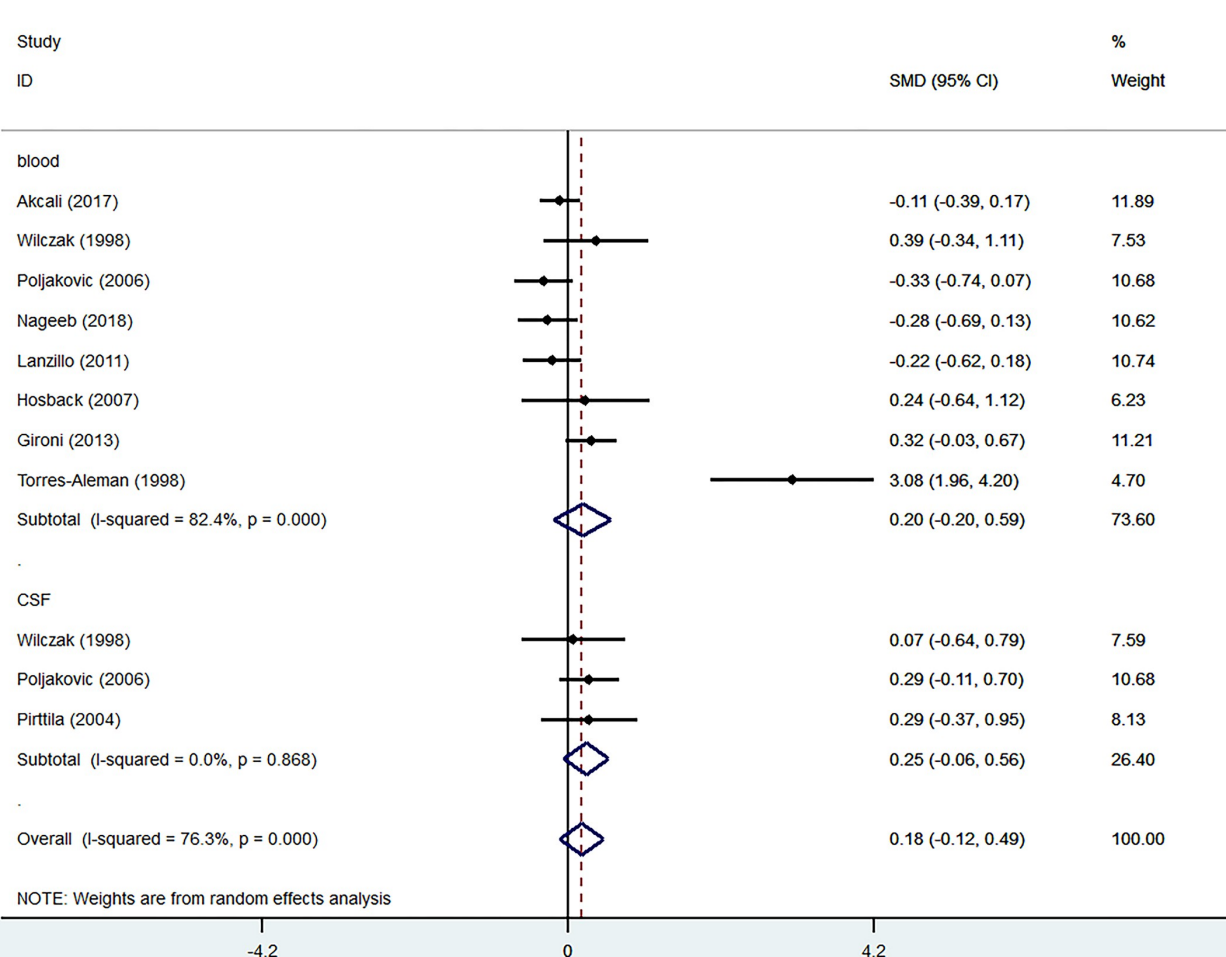
Forest plot of the levels of IGF-1 in MS patients. In this plot, the squares are applied to show the mean effect estimate of each paper along with their 95% CI. The size of each square is considered proportional to the weight of the parameter in the meta-analysis and is also demonstrated in a separate column.

Eight studies measured the levels of IGF-1 in the blood of patients and controls, comprising 664 participants, including 322 patients and 342 controls. Blood levels of IGF-1 were not significantly higher in patients than in controls (SMD = 0.20, 95% CI = -0.20 to 0.59, I2 = 82.4%, K = 8, n = 692) (Fig 2).

**Fig 3:**
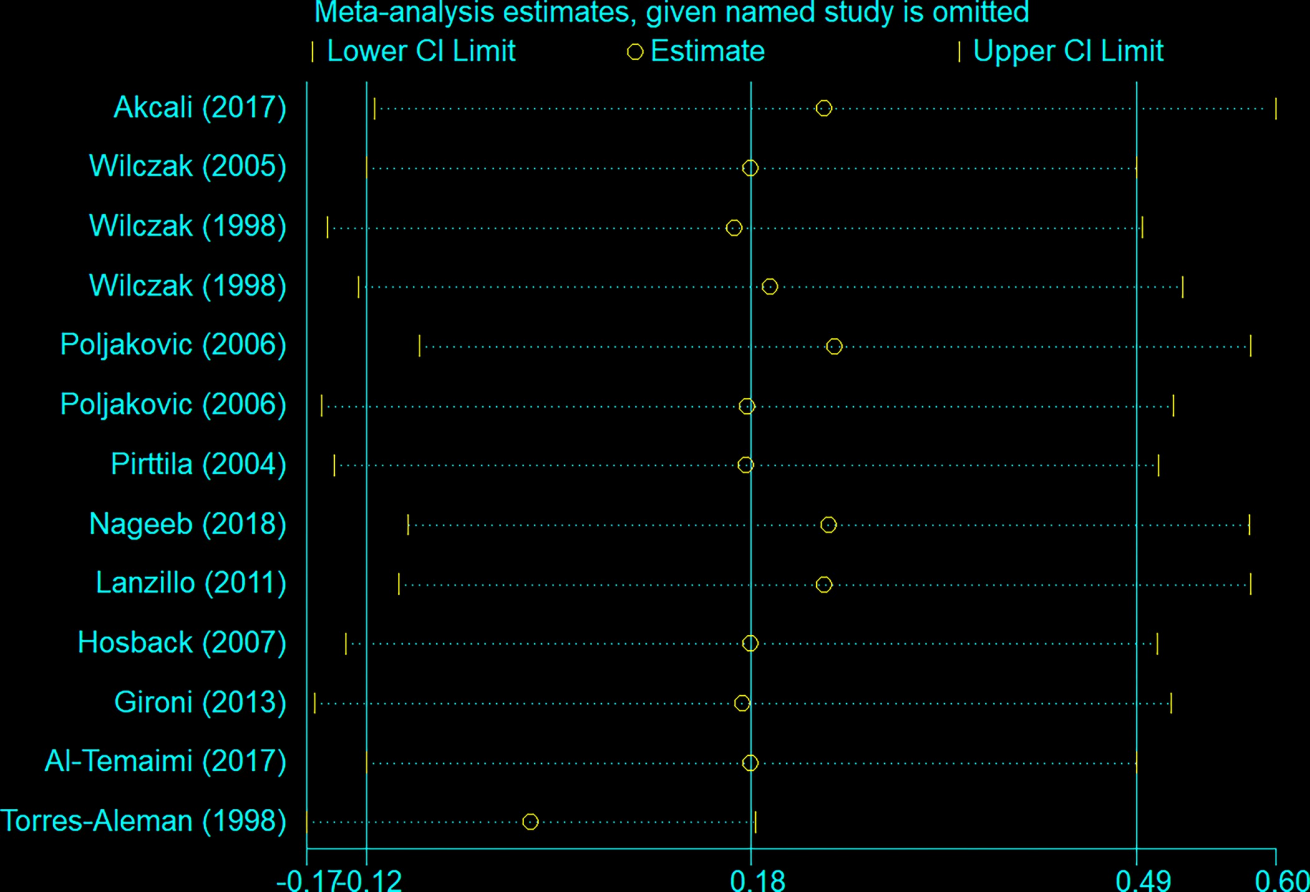
Sensitivity analysis for IGF-1.

A sensitivity analysis for IGF-1 levels in the blood confirmed these non-significant results (Fig 3).

Additionally, three studies investigated the levels of IGF-1 in the CSF of patients and controls, with 164 participants, including 75 patients and 89 controls. CSF levels of IGF-1 were not significantly higher in patients than in controls (SMD = 0.25, 95% CI = -0.06 to 0.56, I2 = 0.0%, K = 3 n = 164) (Fig 2).

#### GH

Three studies [12–14] investigated the levels of GH in the blood samples of patients and controls, with 241 participants, including 120 patients and 121 controls. Blood levels of GH were not significantly higher in patients than in controls (SMD = 0.08, 95% CI = -0.33 to 0.49, I2 = 77.0% K = 3, n = 421).

**Fig 4:**
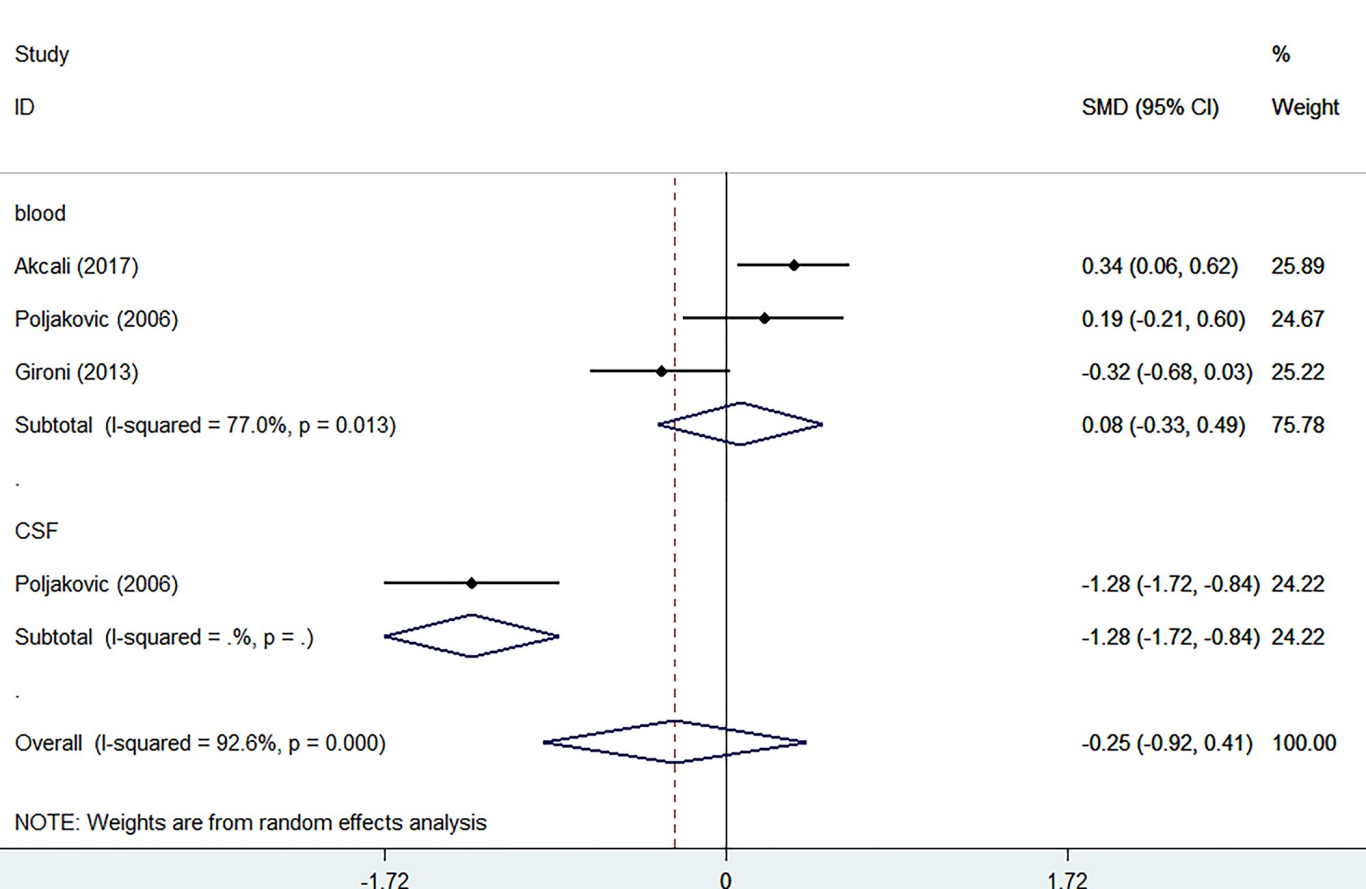
Forest plot of the levels of GH in MS patients. In this plot, the squares are applied to show the mean effect estimate of each paper along with their 95% CI. The size of each square is considered proportional to the weight of the parameter in the meta-analysis and is also demonstrated in a separate column.

However, Poljakovic’s study [14] revealed a significant reduction of GH in the CSF of patients compared to the control group. (p < 0.001) (Fig 4).

#### IGFBP-1

Four studies[15–18] investigated the levels of IGFBP-1 in the blood of the patients and controls, with 255 participants, including 140 patients and 115 controls. The blood levels of IGFBP-1 were significantly higher in patients than in controls (SMD = 0.70, 95% CI = 0.01 to 1.40, I2 = 77%, K = 4, n = 255).

**Fig 5:**
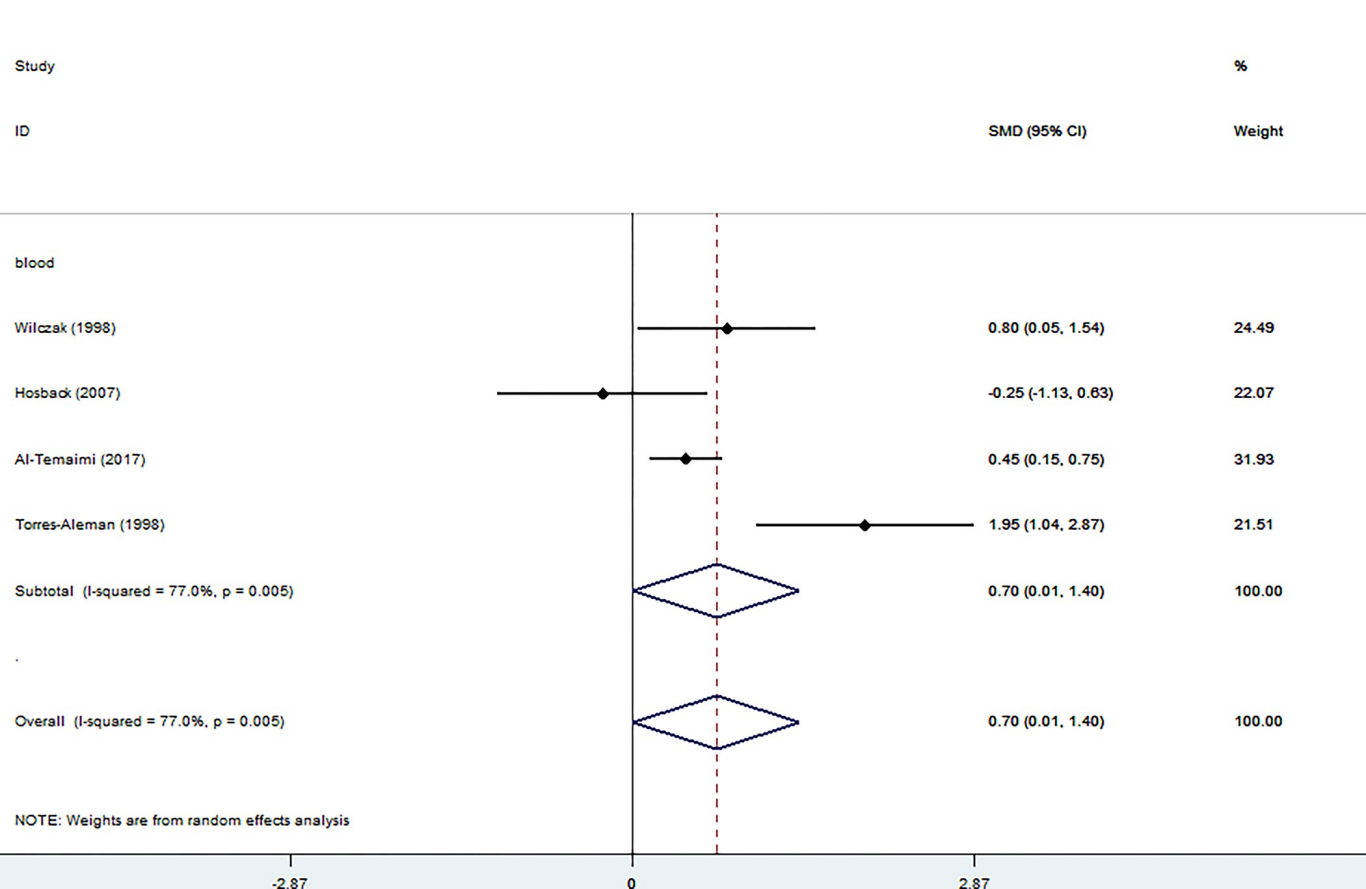
Forest plot of the levels of IGFBP-1 in MS patients. In this plot, the squares are applied to show the mean effect estimate of each paper along with their 95% CI. The size of each square is considered proportional to the weight of the parameter in the meta-analysis and is also demonstrated in a separate column.

However, Al_Temaimi et al. [17] and Torres_Aleman et al.’s [18] studies showed a significant increase in IGFBP-1 in the blood of the patient group compared to the control group (Fig 5).

#### IGFBP-2

Three studies investigated the levels of IGFBP-2 in blood of the patients and controls, including 78 participants, with 40 patients and 38 controls.

The blood levels of IGFBP-2 were not significantly higher in patients than in controls (SMD = 0.43, 95% CI = -0.34 to 1.21, I2 = 64.2%, K = 3, n = 78).

**Fig 6:**
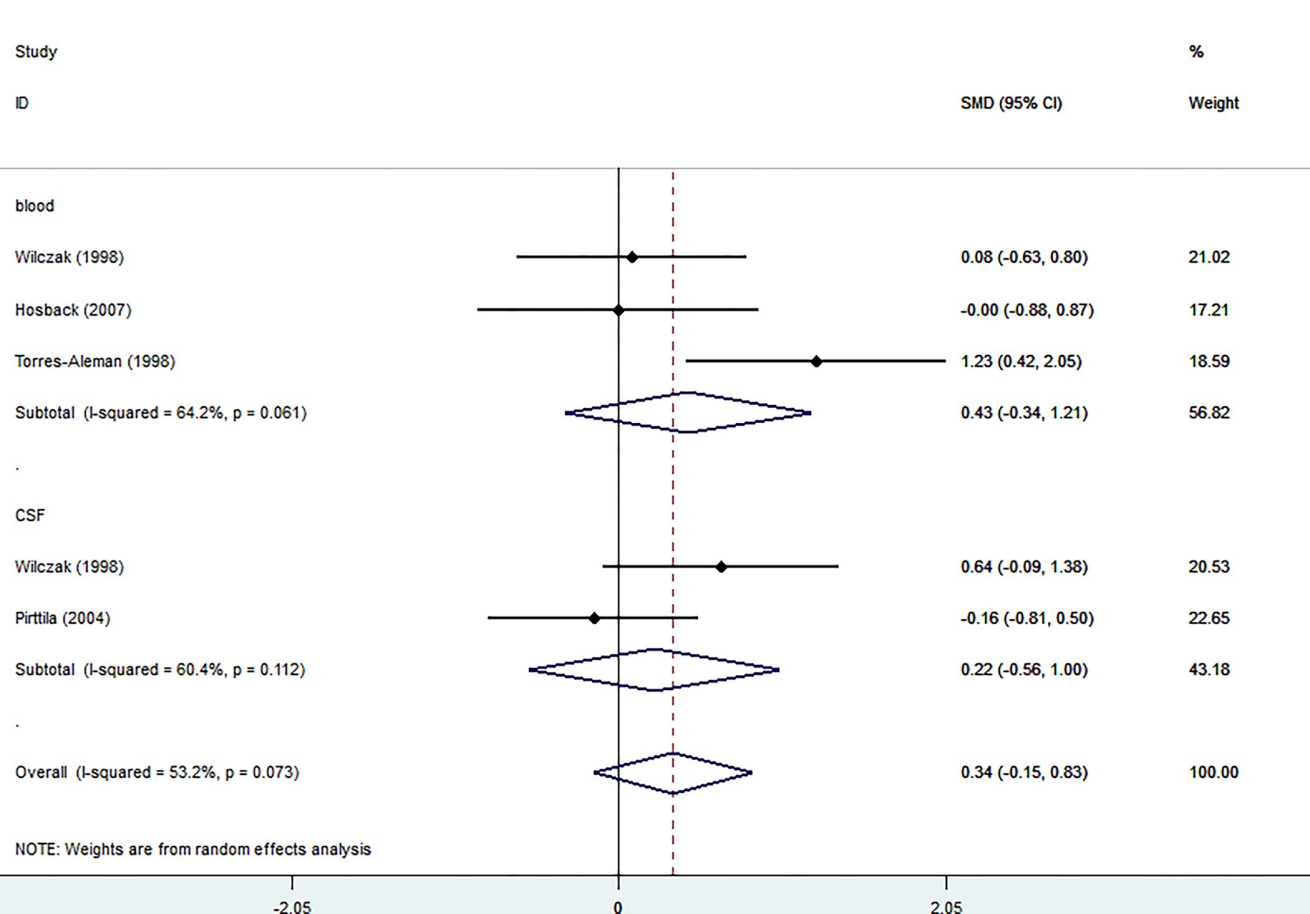
Forest plot of the levels of IGFBP-2 in MS patients. In this plot, the squares are applied to show the mean effect estimate of each paper along with their 95% CI. The size of each square is considered proportional to the weight of the parameter in the meta-analysis and is also demonstrated in a separate column.

Torres_Aleman et al.’s study showed a significant increase in IGFBP-2 levels in the blood of the patient group compared to the control group. Wilkzac and Pritilla’s studies did not show a considerable rise in IGFBP-2 in CSF of the patient group compared to the control group (Fig 6).

#### IGFBP-3

**Fig 7:**
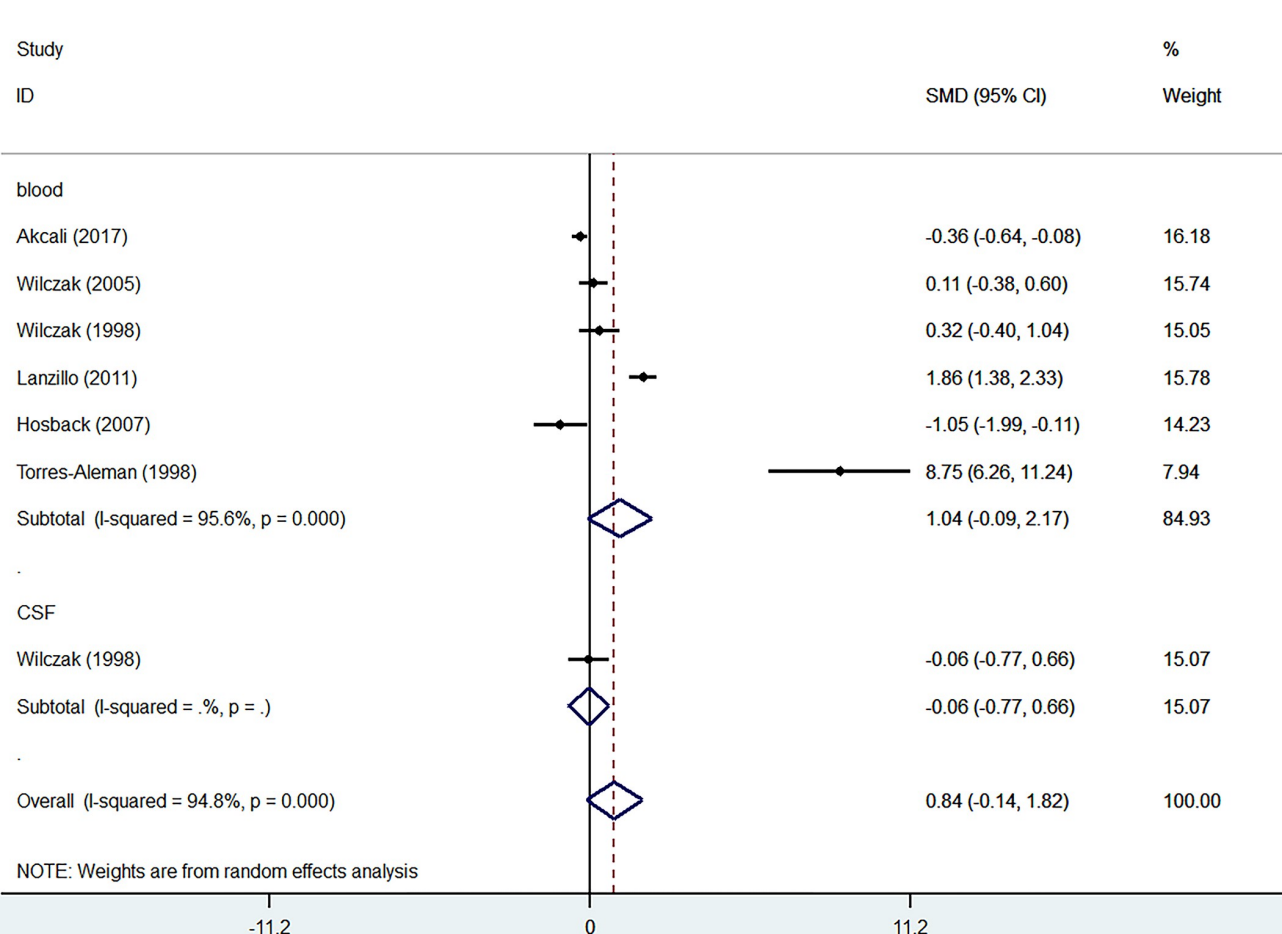
Forest plot of the levels of IGFBP-3 in MS patients. In this plot, the squares are applied to show the mean effect estimate of each paper along with their 95% CI. The size of each square is considered proportional to the weight of the parameter in the meta-analysis and is also demonstrated in a separate column.

Six studies investigated the level of IGFBP-3 in the blood of the patients and controls comprising 443 participants, including 215 patients and 228 controls. Blood levels of IGFBP-3 were not significantly higher in patients than in controls (SMD = 1.04, 95% CI = -0.09 to 2.17, I2 = 95.6%, K = 6, n = 443) (Fig 7).

**Fig 8:**
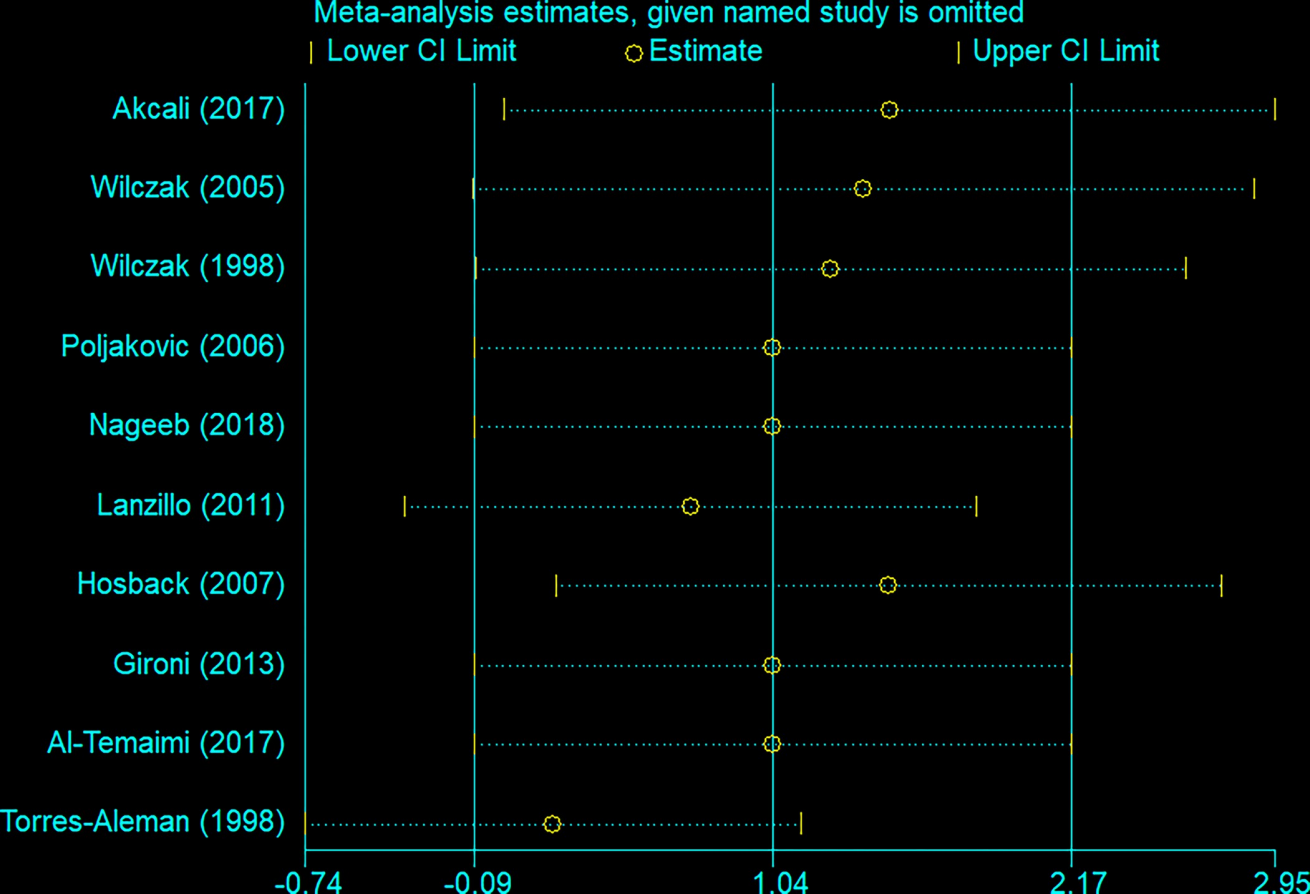
Sensitivity analysis for IGF-BP3.

Sensitivity analysis indicated that removing Hosback et al.’s study resulted in significantly higher levels of IGFBP-3 in patients’ blood than in controls (SMD = 1.47, 95%CI = 0.21 to 2.74) (Fig 8).

Lanzillo et al. [19] and terres_alman et al.’s [18] studies showed a significant increase in levels of IGFBP-3 in blood of the patient group compared to the control group.

Akcali et al. [12] and Hosback et al.’ s [16] studies showed a significant decrease in IGFBP-3 levels in the patients’ blood compared to the control group. However, Wilczac et al.’s [15] study did not show a considerable increase in IGFBP-3 levels in CSF of the patients compared to controls (Fig 7).

Table 2 shows results of the meta-analysis in brief.

**Table 2:** The results of the meta-analysis. Abbreviations: CSF, Cerebrospinal fluid; SMD, standardized mean difference; CI, confidence interval; IGF, insulin-like growth factor; IGFBP, Insulin-like growth factor binding protein; GH, growth hormone.

Factors	Materials	Number of studies	SMD	95%CI	I^2^(%)	P value for heterogenicity
**IGFBP-3**	blood	6	1.04	(-0.09, 2.17)	95.6	0.000
**IGFBP-2**	blood	3	0.43	(-0.34, 1.21)	64.2	0.061
**IGFBP-1**	blood	4	0.70	(0.01, 1.40)	77.0	0.005
**IGF-1**	blood	8	0.20	(-0.20, 0.59)	82.4	0.000
**IGF-1**	CSF	3	0.25	(-0.06, 0.56)	0.0	0.868
**GH**	blood	3	0.08	(-0.33, 0.49)	77	0.013

### Publication bias

**Fig 9:**
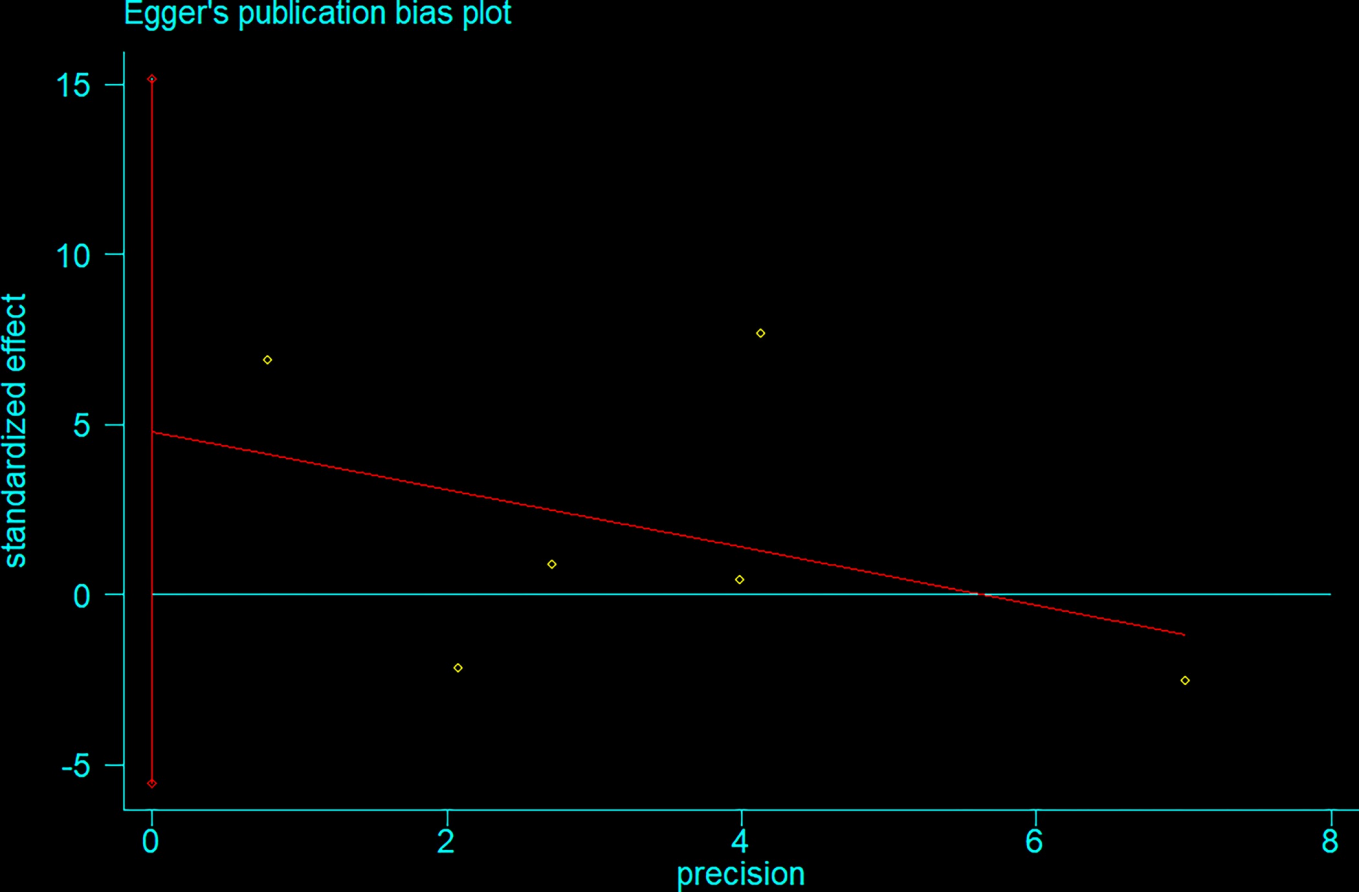
Egger’s plot for publication bias.

We conducted Egger’s test to assess the publication bias. Its result showed no signs of publication bias for IGF-1 (p = 0.40) (Fig 9).

This study was approved by the Iranian National Committee for Ethics in Biomedical Sciences (Code of Ethics: IR.SBMU.RETECH.REC.1400.1117).

## Discussion

A promising approach for MS disease treatment is enhancing the remyelination of exposed axons, in which oligodendrocytes help to maintain neurons by restoring the myelin over the axons; however, this process is reduced as MS disease progresses in this competence [20]. GH, the primary regulator of IGF-1 synthesis, is known to positively affect remyelination and neuronal repair mechanisms, suggesting its prominent role as a neuroprotective agent [13]. IGFBPs, as central players in regulating cell growth and survival, are also responsible for the recruitment and transfer of IGF-1 to the site of target cells and its presentation to cell production receptors [21, 22]. IGF-1 and IGFBPs are believed to have a neuroprotective role in the remyelination process, oligodendrocyte proliferation, neuronal maintenance, and survival in the nervous system. While IGFBP-3 is considered the primary responsible agent in transferring IGF-1 in the blood, IGFBP-2 is likely to be primarily involved in the human CNS [23]. IGFBP-3 was also suggested to reduce IGF-1 bioavailability, influencing neurodegeneration and MS disease progression [19, 24]. Increased levels of IGFBP1 and IGF-1 at the site of target cells induce healing and repairing of cells through myelin synthesis and oligodendrocyte survival, resulting in reduced inflammation in this region [25]. Considering the potential effects of these biomarkers in myelin regeneration and oligodendrocyte survival, these potent factors may be the key to treating neurodegenerative diseases, including MS [25, 26].

### GH

Previous studies showed no difference in serum GH levels between MS patients and healthy controls [12, 14]. Nonetheless, Poljakovic et al. [14] reported reduced levels of GH in CSF of MS patients in comparison to healthy controls, and Gironi et al. [13] found lower GH levels in more severe MS patients during their first ten years of the disease. They also reported slightly reduced GH levels in MS females compared to the healthy control. As mentioned in most studies, there was no statistically significant difference in serum GH and IGF-1 levels between the patient and and control groups [12–14]; however, aging is determined as a considerable risk factor changing the GH, IGF-1, and IGFBP-3 levels in both the MS group and the healthy control and some studies have demonstrated a negative correlation between serum IGF-1 levels with the age of MS patients [12, 13, 19, 27].

### IGF-1

While most previous studies [12, 13, 19, 27] found that serum IGF-1 levels showed no significant difference between MS patients and the healthy control group, Nageeb et al. [27] mentioned significantly lower serum IGF-1 levels in MS patients, considering disease duration of more than five years, primary progressive type MS disease, and more incidences of relapses causing more disability in patients as possible confounders. Moreover, Gironi, et al. [13] reported lower IGF-1 levels in patients older than 50 than the younger group.

In Hosback et al. [16] study, significantly increased levels of IGF-1 were observed in MS patients under treatment by interferon β (INF-β) compared to untreated ones; however, these findings were inconsistent with (Lanzillo et al. 2011; Nageeb, Hashim, and Fawzy 2018) studies that reported no statistically significant difference in serum IGF-1 levels of MS patients receiving INF-β therapy compared to untreated individuals. Moreover, a recently published study by Shahbazi et al. [28] found a significant difference in IGF-1 expression between MS patients and the healthy controls, possibly due to gene polymorphisms differences, as patients carrying the genotype T/T showed higher serum levels of IGF-1 than other individuals carrying C/Tor C/C genotypes; however, based on previous [14, 15, 18] findings, there was no significant difference in CSF or serum IGF-1 levels between MS patients and healthy controls. While they also failed to indicate an association between serum IGF-1 levels and IGFBP-1, -2, and -3 levels between MS patients and the healthy controls, a significant increase in serum levels of IGF-1 (52%), IGFBP-2 (86%), and IGFBP-3 (18%) were yielded in Hosback et al. ‘s findings comparing MS patients to the control group [16]. Another study [12] also reported a positive association between low levels of IGF-1 and IGFBP-3 in MS patients. Our analysis of serum IGF-1 levels considering all eight studies of 664 participants showed no significant difference in serum IGF-1 levels between the MS patients and the healthy controls (Fig 2).

### IGFBP-1

According to our analysis the blood levels of IGFBP-1 were significantly higher in MS patients than in controls. Al-Temaimi et al. [17] mentioned that female MS patients are more prone to have higher serum IGFBP-1 levels than Male types, this elevated biomarker failed to find any association with MS disease progression or the remyelination phase. In contrast, increased serum IGFBP1 level was described as essential to myelin synthesis and oligodendrocyte survival in prior studies [21, 22, 25].

On the other hand, some studies have also shown that in MS, there is an increase in the expression of IGFBP-1 within the oligodendrocytes near chronic active lesion [25, 29]. Upregulation of IGFBP-1, along with similar alterations in other IGF-binding proteins within MS lesions, might contribute to the hindered process of remyelination. This hindrance occurs due to the potential restriction of IGF-1’s crucial actions involved in myelin production [29]. Furthermore, the study conducted by Ye and colleagues highlighted that myelin production was inhibited when IGFBP-1 was upregulated in transgenic mice [30].

Moreover, Bove et al. [31] noted that serum IGFBP1 levels are decreased during the post-menopausal phase in MS females leading to worsening patient symptoms compared to MS male individuals at the same age.

Our analysis of serum IGFBP-1 levels considering all four studies of 255 individuals, showed no significant difference in serum IGFBP-1 levels between the MS group and the healthy control (Fig 4).

### IGFBP-2

The serum levels of IGFBP-2 between MS patients and the control group were investigated by Torres-Aleman et al., Hosback et al., and Wilczak et al. [15, 16, 18] studies, in which they failed to demonstrate any significant difference between the groups. Inconsistent with these results, [15, 32] reported no significant association between MS patients and the healthy group regarding CSF levels of IGFBP-2, however, a significant correlation between CSF IGFBP-2 levels and the age of individuals in the control group was revealed by Pirttilä et al. [32] study. It seems that the result of CSF protein level’s correlation with IGFBP-2 concentrations in MS patients is the only considerable outcome on this matter. Moreover, Torres-Aleman et al. [18] reported elevated serum IGFBP-2 values only related to MS patients undergoing INF-β treatment and ALS individuals. These findings suggest that IGFBP-2 levels may not be a valuable marker for investigating MS remyelination.

### IGFBP-3

Previous studies reported no significant difference in serum IGF-1 and IGFBP-3 levels regarding MS patients and healthy controls [15, 18]. While elevated levels of IGFBP-3 in the Hosback et al. [16] study was only observed in MS patients under INF-β treatment, reduced values of IGFBP-3 were mentioned in patients with severe stages of MS and with a high index of disability in Akcali et al. and Wilczak et al. [12, 24] studies, respectively.

Wilczak et al. [24] also suggested that altered serum IGFBP-3 levels might be a factor correlated with the scale of neurodegeneration and disease progression rate in patients with MS. Prior findings also emphasized on the role of IGFBP-3 in neuronal degeneration by elevation of IGFBP-3 activity in the brains of patients with Alzheimer’s disease. Although Lanzillo et al. [19] study revealed no significant difference in serum IGF-1 levels between MS patients and healthy controls, lower IGF-1/IGFBP-3 ratio and higher levels of IGFBP-3 were reported in their findings, possibly due to lowered IGF-1 bioavailability values for remyelination in more severe-stage MS patients with higher disability rate at ten years of the disease course.

Our analysis of serum IGFBP-3 levels considering all studies demonstrated a significant increase in serum IGFBP-3 levels reported in Lanzillo et al. [19] study, a significantly reduced serum IGFBP-3 levels in Akcali et al. and Wilczak et al. [12, 24] studies, and no difference noted in Al-Temaimi et al., Poljakovic et al., Gironi et al., and Nageeb et al. [13, 14, 17, 27] studies considering the MS group and the healthy control (Fig 8).

The main strength of our study was conducting the first systematic review and meta-analysis comparing the levels of IGF-1, GH, IGFBP-2, and IGFBP-3 between MS patients and healthy controls. However, we were faced with several limitations including the low number of eligible studies for meta-analysis. Moreover, the studies needed to be more sufficient for subgroup meta-analysis or meta-regression for further evaluation.

## Conclusion

In conclusion, no significant difference was detected in serum IGF-1, GH, IGFBP-2 or IGFBP-3 levels between the MS group and healthy control, except for IGFBP-1 which was higher in MS cases than controls. However, IGFBP-3 levels in minor studies correlated with MS patients. These findings suggest that IGFBP-1 could be potentially a determining biomarker in MS. Although, the other mentioned agents may not be considered central determining factors in MS. Further surveys are required to assess this concept more accurately and conduct a more comprehensive meta-analysis.

## References

[1] Chang A., et al., Premyelinating oligodendrocytes in chronic lesions of multiple sclerosis. New England Journal of Medicine, 2002. 346(3): p. 165–173. doi: 10.1056/NEJMoa010994 11796850

[2] Weinstein D.R., Owens G.M., and Gandhi A., Multiple Sclerosis: Systemic Challenges to Cost-Effective Care. Am Health Drug Benefits, 2022. 15(1): p. 13–20. doi: 10.1177/17562864211006499 35586614 PMC9038003

[3] Bishop M. and Rumrill P.D., Multiple sclerosis: Etiology, symptoms, incidence and prevalence, and implications for community living and employment. Work, 2015. 52(4): p. 725–734. doi: 10.3233/WOR-152200 26639011

[4] García-Segura L.M., et al., Localization of insulin-like growth factor I (IGF-I)-like immunoreactivity in the developing and adult rat brain. Brain research, 1991. 560(1–2): p. 167–174. doi: 10.1016/0006-8993(91)91228-s 1722132

[5] Chesik D., Wilczak N., and De Keyser J., The insulin‐like growth factor system in multiple sclerosis. International review of neurobiology, 2007. 79: p. 203–226. doi: 10.1016/S0074-7742(07)79009-8 17531843

[6] Nyberg F. and Burman P., Growth hormone and its receptors in the central nervous system–location and functional significance. Hormone research in paediatrics, 1996. 45(1–2): p. 18–22. doi: 10.1159/000184753 8742113

[7] Wasinski F., Frazão R., and Donato J., Effects of growth hormone in the central nervous system. Archives of endocrinology and metabolism, 2020. 63: p. 549–556. 10.20945/2359-3997000000184 PMC10522235 31939479

[8] Nyberg F. and Hallberg M., Growth hormone and cognitive function. Nature Reviews Endocrinology, 2013. 9(6): p. 357–365. doi: 10.1038/nrendo.2013.78 23629538

[9] Noguchi T., Sugiasaki T., and Tsukada Y., Microcephalic cerebrum with hypomyelination in the growth hormone-deficient mouse (lit). Neurochemical research, 1985. 10(8): p. 1097–1106. doi: 10.1007/BF00965884 2414679

[10] Waters M. and Blackmore D., Growth hormone (GH), brain development and neural stem cells. Pediatric endocrinology reviews: PER, 2011. 9(2): p. 549–553. 22397139

[11] Bianchi V.E., Locatelli V., and Rizzi L., Neurotrophic and neuroregenerative effects of GH/IGF1. International journal of molecular sciences, 2017. 18(11): p. 2441. doi: 10.3390/ijms18112441 29149058 PMC5713408

[12] Akcali A., Bal B., and Erbagci B., Circulating IGF-1, IGFB-3, GH and TSH levels in multiple sclerosis and their relationship with treatment. Neurol Res, 2017. 39(7): p. 606–611. doi: 10.1080/01616412.2017.1321711 28460598

[13] Gironi M., et al., Growth hormone and disease severity in early stage of multiple sclerosis. Mult Scler Int, 2013. 2013: p. 836486. doi: 10.1155/2013/836486 24260717 PMC3821914

[14] Poljakovic Z., et al., Growth hormone and insulin growth factor-I levels in plasma and cerebrospinal fluid of patients with multiple sclerosis. Clinical Neurology and Neurosurgery, 2006. 108(3): p. 255–258. doi: 10.1016/j.clineuro.2005.11.014 16386830

[15] Wilczak N., et al., Insulin-like growth factor system in serum and cerebrospinal fluid in patients with multiple sclerosis. Neuroscience letters, 1998. 257(3): p. 168–170. doi: 10.1016/s0304-3940(98)00829-5 9870347

[16] Hosback S., et al., Circulating insulin-like growth factors and related binding proteins are selectively altered in amyotrophic lateral sclerosis and multiple sclerosis. Growth Horm IGF Res, 2007. 17(6): p. 472–9. doi: 10.1016/j.ghir.2007.06.002 17697791

[17] Al-Temaimi R., et al., Remyelination modulators in multiple sclerosis patients. Exp Mol Pathol, 2017. 103(3): p. 237–241. doi: 10.1016/j.yexmp.2017.11.004 29108879

[18] Torres-Aleman I., Barrios V., and Berciano J., The peripheral insulin-like growth factor system in amyotrophic lateral sclerosis and in multiple sclerosis. Neurology, 1998. 50(3): p. 772–776. doi: 10.1212/wnl.50.3.772 9521273

[19] Lanzillo R., et al., Insulin-like growth factor (IGF)-I and IGF-binding protein-3 serum levels in relapsing-remitting and secondary progressive multiple sclerosis patients. European Journal of Neurology, 2011. 18(12): p. 1402–1406. doi: 10.1111/j.1468-1331.2011.03433.x 21585623

[20] Compston A. and Sadovnick A.D., Epidemiology and genetics of multiple sclerosis. Current Opinion in Neurology, 1992. 5(2): p. 175–181. 1623245

[21] Mason J., et al., Insulin-like growth factor-1 inhibits mature oligodendrocyte apoptosis during primary demyelination. Journal of Neuroscience, 2000. 20(15): p. 5703–5708. doi: 10.1523/JNEUROSCI.20-15-05703.2000 10908609 PMC6772563

[22] Ribbons K.A., et al., Male sex is independently associated with faster disability accumulation in relapse-onset MS but not in primary progressive MS. PloS one, 2015. 10(6): p. e0122686. doi: 10.1371/journal.pone.0122686 26046348 PMC4457630

[23] Rotwein P., Folz R., and Gordon J., Biosynthesis of human insulin-like growth factor I (IGF-I). The primary translation product of IGF-I mRNA contains an unusual 48-amino acid signal peptide. Journal of Biological Chemistry, 1987. 262(24): p. 11807–11812. 3624235

[24] Wilczak N., et al., Serum levels of insulin-like growth factor-1 and insulin-like growth factor binding protein-3 in relapsing and primary progressive multiple sclerosis. Multiple Sclerosis Journal, 2005. 11(1): p. 13–15. doi: 10.1191/1352458505ms1123oa 15732261

[25] Wilczak N., et al., IGF binding protein alterations on periplaque oligodendrocytes in multiple sclerosis: implications for remyelination. Neurochemistry international, 2008. 52(8): p. 1431–1435. doi: 10.1016/j.neuint.2008.03.004 18471934

[26] Chesik D., Wilczak N., and De Keyser J., IGF-1 regulates cAMP levels in astrocytes through a β2-adrenergic receptor-dependant mechanism. International journal of medical sciences, 2008. 5(5): p. 240. 18690292 10.7150/ijms.5.240 PMC2500150

[27] Nageeb R.S., Hashim N.A., and Fawzy A., Serum insulin-like growth factor 1 (IGF-1) in multiple sclerosis: relation to cognitive impairment and fatigue. The Egyptian Journal of Neurology, Psychiatry and Neurosurgery, 2018. 54(1): p. 1–8. 30294204 10.1186/s41983-018-0026-y PMC6153711

[28] Shahbazi M., et al., Novel functional polymorphism in IGF-1 gene associated with multiple sclerosis: a new insight to MS. Multiple sclerosis and related disorders, 2017. 13: p. 33–37. 28427698 10.1016/j.msard.2017.02.002

[29] Chesik D., et al., Insulin‐like growth factor binding protein‐1 activates integrin‐mediated intracellular signaling and migration in oligodendrocytes. Journal of neurochemistry, 2010. 113(5): p. 1319–1330. doi: 10.1111/j.1471-4159.2010.06703.x 20345750

[30] Ye P., Carson J., and D’Ercole A.J., In vivo actions of insulin-like growth factor-I (IGF-I) on brain myelination: studies of IGF-I and IGF binding protein-1 (IGFBP-1) transgenic mice. Journal of Neuroscience, 1995. 15(11): p. 7344–7356. doi: 10.1523/JNEUROSCI.15-11-07344.1995 7472488 PMC6578047

[31] Bove R., et al., Exploration of changes in disability after menopause in a longitudinal multiple sclerosis cohort. Multiple Sclerosis Journal, 2016. 22(7): p. 935–943. doi: 10.1177/1352458515606211 26447063 PMC4824677

[32] Pirttilä T., et al., Cerebrospinal fluid insulin‐like growth factor‐1, insulin growth factor binding protein‐2 or nitric oxide are not increased in MS or ALS. Acta neurologica scandinavica, 2004. 109(5): p. 337–341. doi: 10.1111/j.1600-0404.2004.00223.x 15080860

